# Oxidation of Cathepsin D by Hydroxy Radical: Its Effect on Enzyme Structure and Activity against Myofibrillar Proteins Extracted from *Coregonus peled*

**DOI:** 10.3390/molecules28135117

**Published:** 2023-06-29

**Authors:** Mengjie Ma, Pingping Liu, Chaoye Wang, Xiaorong Deng, Lianfu Zhang, Jian Zhang

**Affiliations:** 1 Key Laboratory of Agricultural Product Processing and Quality Control of Specialty (Co-Construction by Ministry and Province), School of Food Science and Technology, Shihezi University, Shihezi 832003, China; 20202011004@stu.shzu.edu.cn (M.M.); liupp0222@163.com (P.L.); 20202111024@stu.shzu.edu.cn (C.W.); dxr20099@163.com (X.D.); 2 Key Laboratory of Food Nutrition and Safety Control of Xinjiang Production and Construction Crops, School of Food Science and Technology, Shihezi University, Shihezi 832003, China; 3 State Key Laboratory of Food Science and Technology, School of Food Science and Technology, Collaborative Innovation Center of Food Safety and Quality Control in Jiangsu Province, Jiangnan University, Wuxi 214122, China

**Keywords:** cathepsin D, myofibrillar protein, protein oxidation, *Coregonus peled*

## Abstract

In this study, cathepsin D was oxidized in vitro with different concentrations of H_2_O_2_, and the activity, structure, and extent of myofibrillar protein degradation by oxidized cathepsin D were evaluated. The sulfhydryl content of cathepsin D decreased to 9.20% after oxidation, while the carbonyl content increased to 100.06%. The β-sheet in the secondary structure altered due to oxidation as well. The changes in the intrinsic fluorescence and UV absorption spectra indicated that oxidation could cause swelling and aggregation of cathepsin D molecules. The structure of cathepsin D could change its activity, and the activity was highest under 1 mM H_2_O_2_. Cathepsin D could degrade myofibrillar proteins in different treatment groups, and the degree of degradation is various. Therefore, this study could provide a scientific basis for the mechanism of interaction among hydroxyl radical oxidation, cathepsin D, and MP degradation.

## 1. Introduction

Meat and aquatic products are important consumables in people’s daily lives. However, after animals are slaughtered, a series of changes occur due to the interaction of physiology, chemistry, biochemistry, and microorganisms [1]. *Coregonus peled*, a cold-water fish in Xinjiang, is rich in high-protein, high-fat, various amino acids, minerals, vitamins, and unsaturated fatty acids, making it a popular choice among consumers both domestically and abroad in recent years [2]. Unfortunately, the production and processing of *Coregonus peled* are limited due to its immediate death out of water, which can trigger autolysis and affect processing and marketing. Even if frozen *Coregonus peled* is brought back to room temperature, tissue autolysis still occurs. The degradation of protein skeleton by mitochondrial apoptotic enzymes, calpain, and cathepsin after fish death leads to meat softening.

The internal environment of the fish body collapses after death, and during the late stage of rigor, lysosomes rupture, intracellular pH decreases, and cathepsins are released into the cellular matrix [3]. Cathepsin D is one of the major aspartyl cathepsins found in muscles of fish, and its activity is several times higher than that found in mammals [4]. During processing, cathepsin diffusion can occur in fish products [5]. Due to its activity at pH 3.0–4.5, cathepsin D, an aspartic protease, is an important lysosomal acid protease [6]. Initially synthesized as a glycoprotein modified by man-nose 6-phosphate, cathepsin D is transported to the Golgi apparatus through lysosome-targeted receptors, where it is transferred to the lysosome and processed into a stable and active form [7]. Cathepsins are generally found in lysosomes and the cellular matrix but may be released outside the cell through exocytosis [8]. Interestingly, cathepsin D can cleave and degrade proteins and peptides, activate growth factors and zymogen precursors, and process enzyme activators [9,10]. Postmortem autolysis causes a deterioration of fish quality during processing and storage, leading to adverse changes in sensory properties of the final product [11]. One of the most common phenomena in fish after death is protein oxidation. Oxidation of proteins is generally caused by the oxidative attack of reactive oxygen species and the oxidative modification of amino acid side chains. Muscle tissue is rich in natural components such as hemoglobin, transition metals, and oxidase, which are potential precursors or catalysts for the formation of ROS and play an important role in the oxidation of meat protein [12]. Protein oxidation affects the properties of proteins and thus the quality of fish. Specifically, it results in the modification of amino acid side chains, including all aminoacyl groups of the protein [13]. Examples of these modifications include hydroxylation, chlorination, and nitration of aliphatic amino acid side chains, sulfonation of methionine residues, nitration of sulfhydryl groups, and carbonylation of amino acid side chains [14].

Studying the mechanism of cathepsin D in the tissue softening phenomenon after the death of fish and the effect of cathepsin D on the degradation of myofibrillar protein under the condition of hydroxyl radical oxidation system is of great significance for controlling oxidation in the production process, slowing down the deterioration of muscle texture, and producing high-quality products.

## 2. Results and Discussion

### 2.1. Activity of Cathepsin D in Hydroxyl Radical Oxidation System

Cathepsin D is present in almost all mammalian cells and tissues, and is the earliest discovered and most reported cathepsin [15]. Cathepsins are involved in many cellular activities in vivo, including protein hydrolysis, cell apoptosis regulation, and promotion of tumor cell invasion and metastasis [16,17]. The activity of cathepsin D was measured to determine whether protease oxidation resulted in changes in protease activity.

**Figure 1:**
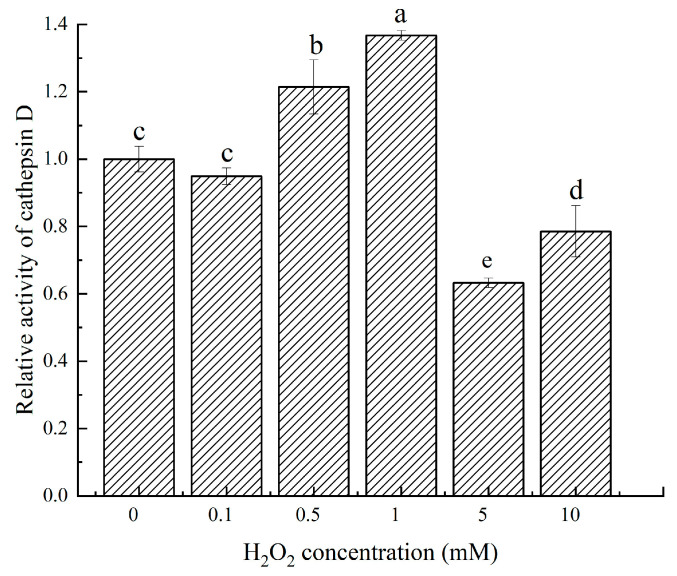
Relative activity of cathepsin D in response to different H_2_O_2_ oxidation concentrations. a–e: different lowercase letters denote significant differences (*p* < 0.05).

The activity of cathepsin D after H_2_O_2_ oxidation at different concentrations was measured by using acidified bovine serum protein as a substrate, and the results were shown in Figure 1. With the increase in oxidation concentration, the activity of cathepsin D generally showed a trend of first increasing and then decreasing. Under the condition of low oxidation concentration, the activity of cathepsin D increased with the increase in oxidation concentration. When the oxidation concentration of H_2_O_2_ was 1 mmol, the activity of cathepsin D was the highest, which increased by 36.74% compared with 0 probably owing to oxidation. The active site of cathepsin D was exposed, and the ability of cathepsin D to bind substrates was enhanced. The reduced activity of cathepsin at high oxidative concentrations may be due to the fact that at high concentrations of H_2_O_2_, the spatial construct of cathepsin D was further altered so that it began to fold, and the active site was hidden inside, thus resulting in reduced activity of cathepsin D.

### 2.2. Changes in the Carbonyl Content of Cathepsin D

**Figure 2:**
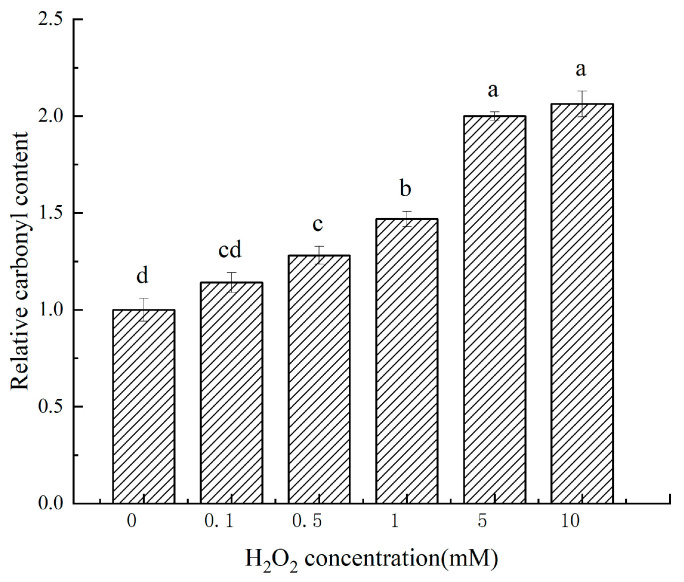
Changes in the carbonyl content of cathepsin D at different H_2_O_2_ oxidation concentrations. a–d: different lowercase letters denote significant differences (*p* < 0.05).

Figure 2 shows that as the concentration of H_2_O_2_ increased, the relative carbonyl content of cathepsin D also gradually increased. When the H_2_O_2_ concentration reached 10 mmol/L, the carbonyl content of cathepsin D increased by 100.06% compared with 0 mmol/L (*p* < 0.05). Carbonyl groups can form through direct oxidation, covalent binding to non-protein carbonyl compounds, cleavage of peptide backbone, and reducing sugar [18]. Protein carbonylation is an irreversible non-enzymatic oxidation reaction, and therefore, protein carbonylation serves as a marker of protein oxidation [19]. Numerous studies have demonstrated that the degree of protein oxidation is directly proportional to the content of carbonyl compounds. In the Fenton system, free radicals are generated through the REDOX reaction of Fe^3+^. Amino acids such as arginine, lysine, and threonine in the amino acid side chain are easily modified and converted to carbonyl groups through dehydrogenation [20]. Furthermore, oxidative cleavage of peptide bonds can also increase the content of carbonyl derivatives [21] In this test, the content of carbonyl groups monotonically increased with the degree of oxidation, which is consistent with the findings of Zhang et al. [11], who studied the treatment of *Coregonus peled* myofibrillar protein by hydroxyl radicals.

### 2.3. Changes in the Sulfhydryl Content of Cathepsin D

**Figure 3:**
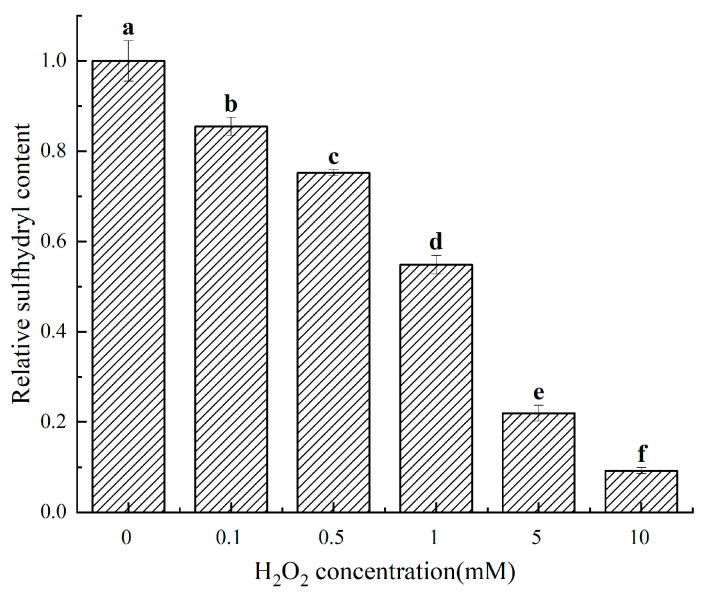
Changes in the relative sulfhydryl content of cathepsin D at different H_2_O_2_ oxidation concentrations. a–f: different lowercase letters denote significant differences (*p* < 0.05).

Figure 3 shows that as the concentration of H_2_O_2_ increased, the sulfhydryl content of the other treatment groups decreased to 85.49%, 75.221%, 54.87%, 21.95%, and 9.20% of the 0 mM treatment group, respectively (*p* < 0.05), indicating a significant effect of H_2_O_2_ on cathepsin D. Sulfhydryl groups in amino acids are susceptible to oxidation, which can lead to complex biochemical reactions resulting in various oxidation products such as sulfonic acid and disulfide bonds. These products make the spatial structure of proteins more compact, leading to a reduction in sulfhydryl group content [22]. In this experiment, the sulfhydryl content of cathepsin D treated with different H_2_O_2_ concentrations decreased monotonously with the increase in oxidation concentration. This phenomenon is consistent with the findings of Zhang et al. [20], who studied the effect of H_2_O_2_ on myofibrillar proteins in pigs. Fu et al. [22] also reported similar results while examining the structural effects of in vitro oxidation of H_2_O_2_ on beef myofibrils.

### 2.4. Structural Changes in Cathepsin D

#### 2.4.1. Ultraviolet Absorption Spectrum Analysis of Cathepsin D

**Figure 4:**
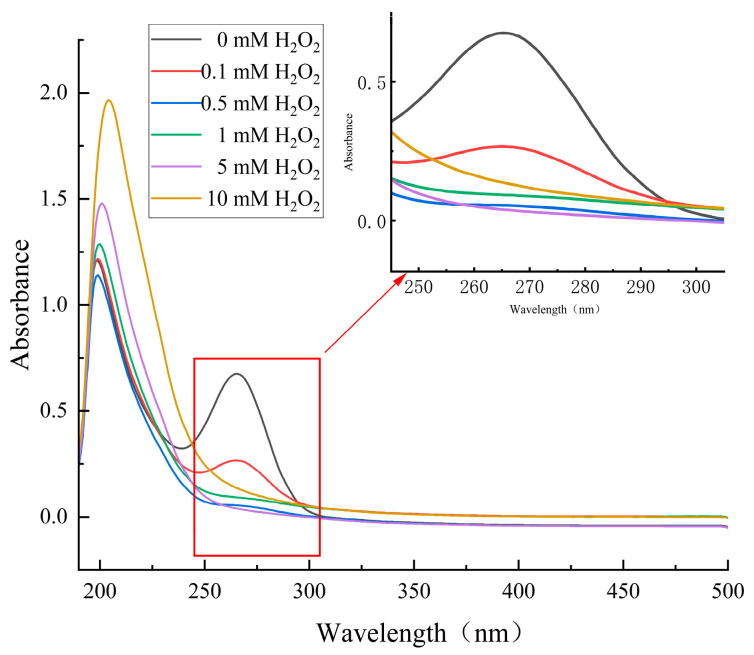
Ultraviolet absorption spectrum of cathepsin D at different H_2_O_2_ oxidation concentrations.

UV absorption spectroscopy is a commonly used technique to assess the oxidative modification of protein aromatic amino acid side chains caused by oxidation, which allows for the evaluation of changes in protein structure [23]. Figure 4 shows the UV absorption spectra of cathepsin D treated with different H_2_O_2_ oxidation concentrations. The enzyme protein exhibited a pronounced absorption peak around 266 nm, which was the characteristic absorption peak of aromatic amino acid residues such as phenylalanine, tyrosine, and tryptophan [24]. As depicted in Figure 4, the characteristic absorption peak of cathepsin D gradually flattened out with increasing oxidation concentration due to oxidative modification of aromatic amino acids. The decrease in the characteristic absorption peak also indicated changes in the secondary and tertiary structure of the enzyme protein. At higher oxidation concentrations, cathepsin D showed almost no absorption peak near 266 nm, indicating that higher concentrations of H_2_O_2_ alter the primary structure of cathepsin D. This finding is consistent with the results of Lv et al. [25], who investigated the effect of 4-hydroxy-2-nonenal treatment on the IgE binding ability and structure of shrimp tropomyosin.

#### 2.4.2. Changes in Endogenous Fluorescence Intensity of Cathepsin D

**Figure 5:**
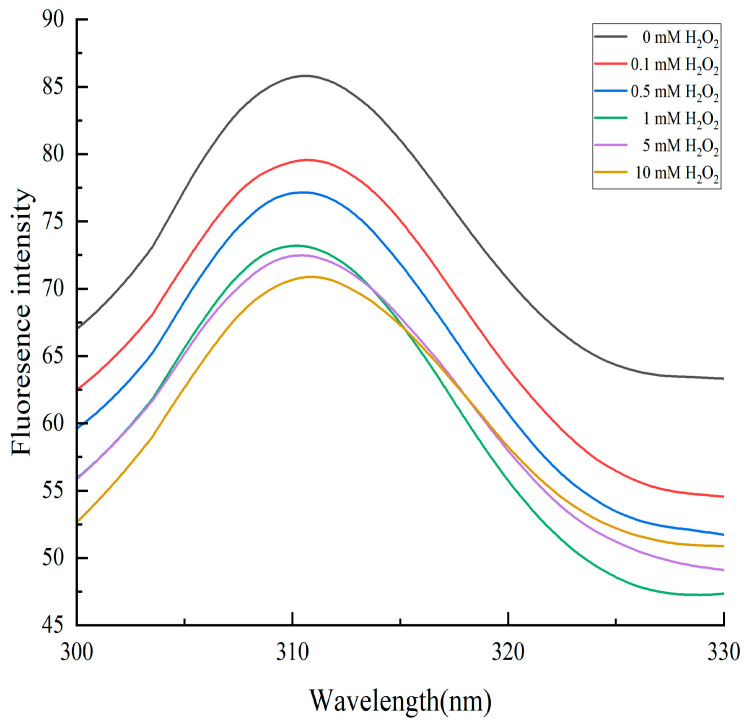
Changes in the fluorescence spectrum of cathepsin D at different H_2_O_2_ oxidation concentrations.

Endogenous fluorescence absorption of proteins is a useful tool to assess changes in protein structure and conformation. Proteins contain fluorescent luminescent groups such as tryptophan, tyrosine, and phenylalanine. Oxidation can trigger structural changes in proteins [26]. Tryptophan is particularly vulnerable to attack by reactive oxygen species, leading to its consumption and subsequent fluorescence quenching. Figure 5 shows that with an increase in H_2_O_2_ oxidation concentration, the fluorescence absorption value of cathepsin D decreased because the tryptophan residue, previously located inside the protein, was exposed to oxidation-induced conformational changes that resulted in fluorescence quenching and reduced fluorescence intensity. This finding is consistent with Zhang et al.’s study [20], which investigated the effect of in vitro oxidation on pork protein. Furthermore, high levels of oxidation can cause fluorescence quenching. As the oxidation concentration increased, the fluorescence of cathepsin D exhibited a blue shift caused by increased steric hindrance resulting from protein aggregation due to oxidation. Guo et al. [27] also reported this fluorescence phenomenon when studying the oxidation of Coregonus peled myofibrillar protein by hydroxyl radicals.

#### 2.4.3. Circular Dichroism (CD) Analysis

**Figure 6:**
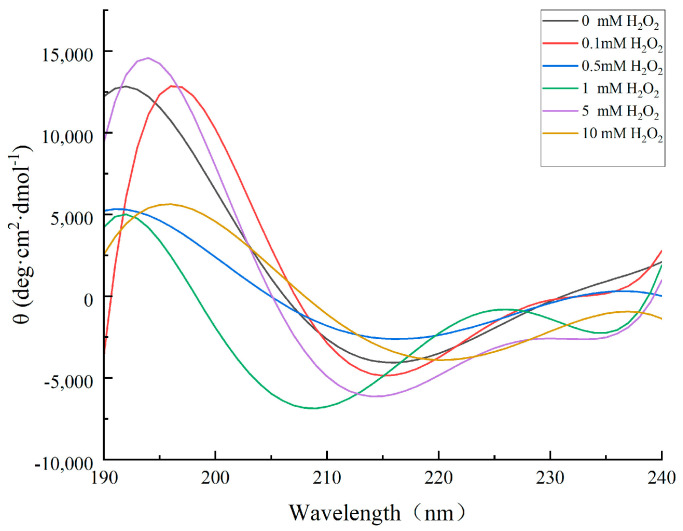
Circular dichroism (CD) of cathepsin D at different H_2_O_2_ oxidation concentrations.

Far-UV CD is a technique used to study the secondary structure of proteins, where peptide bonds serve as chromophores, whereas near-UV CD allows for the evaluation of the tertiary structure of proteins [28]. CD in these wavelength ranges can be used to investigate temporal changes in local and nonlocal structures. The primary structure of a protein refers to its amino acid sequence, while the secondary structure pertains to the spatial arrangement of amino acids. α-helices have hydrogen bonds between residues i and i + 4 on the same chain, β-sheets have hydrogen bonds between adjacent residues, and irregular helices have an uneven arrangement of amino acids. Different secondary structure elements exhibit distinctive far-UV CD spectra. Tertiary structure, which gives proteins their function, refers to their three-dimensional configuration [28]. As depicted in Figure 6, under different concentrations of H_2_O_2_ oxidation, the CD spectrum of cathepsin D exhibited a clear positive peak at 195 nm and a distinct negative peak at 215 nm, indicating that the β-sheet structure was dominant in the cathepsin D group. Like all aspartic proteases, the cathepsin D structure is a double-leaf molecule primarily comprising β-sheets, which is consistent with previous studies on cathepsin D [29,30]. These changes in the CD spectrum were consistent with the peak value of the cathepsin D treated with H_2_O_2_, indicating that the protein conformation of cathepsin D had changed to varying degrees. Oxidation of H_2_O_2_ could alter the secondary structure of cathepsin D and affect its enzyme activity.

### 2.5. Analysis of Myofibrillar Protein Degradation by Oxidized Cathepsin D In Vitro

#### 2.5.1. SDS-PAGE

**Figure 7:**
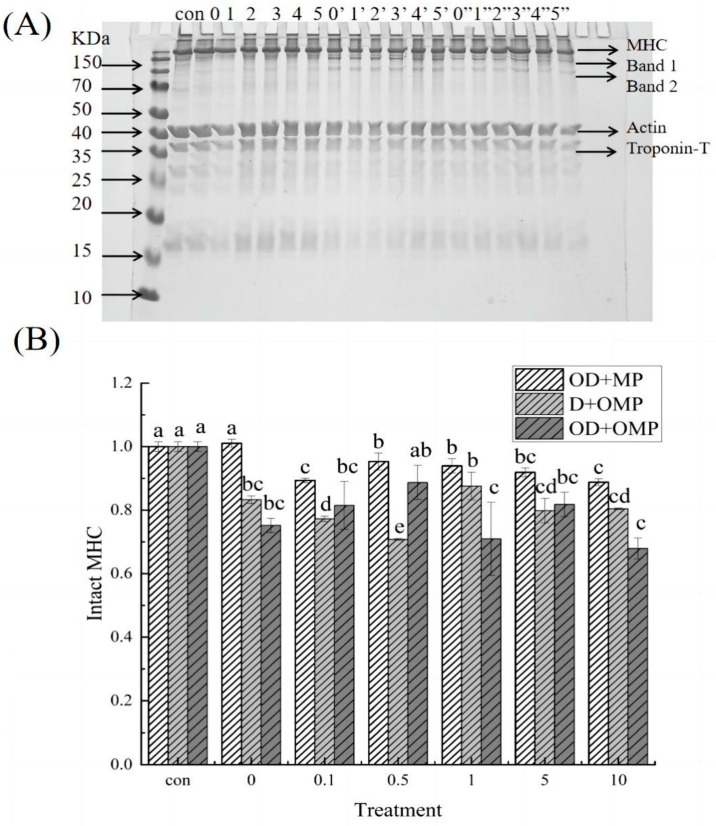
SDS-PAGE profiles of cathepsin D degradation of myofibrillar proteins at different H_2_O_2_ oxidation concentrations. (**A**) SDS electrophoresis of cathepsin D degrading myofibrillar proteins in different treatment groups; (**B**) degradation of MHC in different treatment groups; 0, 1, 2, 3, 4 and 5 representing treatment with 0, 0.1, 0.5, 1, 5, 10 mM H_2_O_2_ oxidized cathepsin D and unoxidized myofibrillar protein incubation groups(OD + MP); 0′, 1′, 2′, 3′, 4′ and 5′ was the group incubated with cathepsin D and 0, 0.1, 0.5, 1, 5, 10 mM H_2_O_2_ oxidized myofibrillar protein (D + OMP); 0″, 1″, 2″, 3″, 4″ and 5″ is the grouping incubated with 0, 0.1, 0.5, 1, 5, and 10 mM H_2_O_2_ oxidized cathepsin D and 0, 0.1, 0.5, 1, 5, and 10 mM H_2_O_2_ oxidized myofibrillar protein (OD + OMP). a–e: different lowercase letters denote significant differences (*p* < 0.05).

In order to evaluate the ability of cathepsin D to degrade myofibrillar proteins of *Coregonus peled* under the influence of hydroxyl radical oxidation system, oxidization of cathepsin D(OD) was performed, or untreated cathepsin D were incubated with oxidization myofibrillar protein (OMP) or non-oxidization myofibrillar protein for 2 h at 4 °C. SDS-PAGE and WB were then used to examine the extent and pattern of cathepsin D degradation of myofibrillar proteins in this treatment group. As shown in Figure 7A, there were significant differences in the degradation of myofibrillar proteins in the three different incubation groups, especially heavy chain myosin with molecular weight above 70 kDa. In the D + OMP group and the OD + OMP group, myosin heavy chain (MHC) degradation bands 1 and 2 were significantly observed, compared with the group that only oxidized cathepsin D. The other two groups had a greater degree of degradation for MHC. The individual components of myofibrillar proteins were analyzed in detail below.

#### 2.5.2. Myosin Heavy Chain (MHC)

According to the solubility of proteins, muscle proteins can be divided into three groups: sarcoplasmic proteins (soluble proteins), myofibrillar proteins (salt-soluble proteins), and meat matrix proteins (insoluble proteins) [31]. Among them, myofibrillar proteins are the most important in meat processing. According to their properties in muscle, they can be divided into three categories: proteins involved in muscle contraction, proteins regulating muscle contraction and cytoskeletal proteins. Myosin, the major protein in skeletal muscle, is a major component of the thick filament of myofibrils with a molecular weight of approximately 510 kDa and includes the myosin heavy chain (MHC) and light chain (MLC). As shown in Figure 7A, there was a significant degradation of MHC in all three incubation treatment groups with increasing oxidative concentration.

Among them, in the OD + MP group, the degradation degree of the MHC band showed a trend of first increasing and then decreasing, which indicated that the MHC caused the greatest degradation of cathepsin D substrate at the oxidation concentration of 10 mM, degraded to 88.82% of the original protein. However, no obvious degradation bands of MHC were observed in this group.

In the D + OMP group, the degree of MHC degradation first increased and then decreased, and the degradation band 1 and band 2 could be clearly observed. It may be due to the fact that the high concentration of H_2_O_2_ caused protein aggregation and reduced the binding site with cathepsin D. At the oxidation concentration of 0.1 mM, the degradation rate of MHC was the highest. It is possible that cathepsin D is a very active protease against native proteins, but it has higher activity against denatured proteins. In the OD + OMP group, cathepsin D and cathepsin D were oxidized at different concentrations, and MHC bands were significantly degraded, and the existence of degraded bands 1 and 2 was also obviously observed, indicating that oxidative modification could cause changes in the protein structure of myofibrillar protein and cathepsin D. Different concentrations of H_2_O_2_ had various effects on myofibrillar protein and enzyme protein. Hence, the degree of oxidized cathepsin D degradation of oxidized myofibrillar protein was also different, among which the degradation degree was greater in the 10 mM H_2_O_2_ treatment group, which was the same as the OD + MP treatment group. In the three different incubation groups, the degradation rate of both myofibrillar protein and enzymatic protein oxidized was significantly higher than that of the group only oxidized by cathepsin D, indicating that oxidation had a significant effect on both myofibrillar protein and enzymatic protein, and oxidation of myofibrillar protein could also accelerate the degradation rate of MHC by cathepsin D. This situation was also true in vivo after fish death, and the grouping of OD + OMP was closer to the real situation of cathepsin D degradation of myofibrillar proteins after fish death. These results demonstrated that the softening of fish tissue could be well inhibited by controlling the appropriate oxidation concentration.

#### 2.5.3. Actin

**Figure 8:**
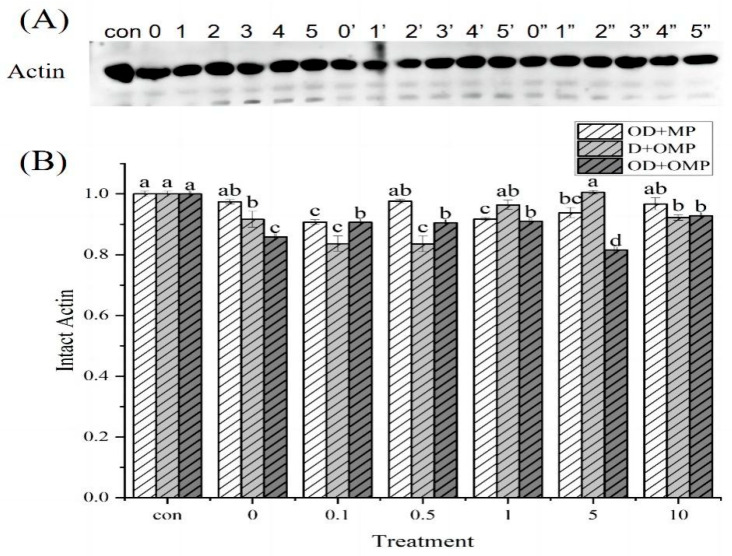
Western-blotting analysis of actin degradation by cathepsin D at different H_2_O_2_ oxidation concentrations. (**A**) WB analysis of actin degradation by cathepsin D in different treatment groups; (**B**) actin degradation in different treatment groups; 0, 1, 2, 3, 4 and 5 representing treatment with 0, 0.1, 0.5, 1, 5, 10 mM H_2_O_2_ oxidized cathepsin D and unoxidized myofibrillar protein incubation groups(OD + MP); 0′, 1′, 2′, 3′, 4′ and 5′ was the group incubated with cathepsin D and 0, 0.1, 0.5, 1, 5, 10 mM H_2_O_2_ oxidized myofibrillar protein (D + OMP); 0″, 1″, 2″, 3″, 4″ and 5″ is the grouping incubated with 0, 0.1, 0.5, 1, 5, and 10 mM H_2_O_2_ oxidized cathepsin D and 0, 0.1, 0.5, 1, 5, and 10 mM H_2_O_2_ oxidized myofibrillar protein (OD + OMP). a–d: different lowercase letters denote significant differences (*p* < 0.05).

Actin is an important component of the cytoskeleton and plays an extremely critical role in eukaryotic cells. The actin cytoskeleton plays a role in the generation and maintenance of cell morphology and polarity, intracellular transport and contractility, motility, and cell division [32]. Actin is the second most abundant protein in the cell and is an essential part of sarcomere filaments. The degradation of actin by cathepsin D treated with different concentrations of H_2_O_2_ was quantified by WB (Figure 8A), and the results are shown in Figure 8B. Degradation bands appeared in three different treatment groups. After 2 h of incubation with oxidized cathepsin D and unoxidized myofibrillar protein (OD + MP), different degrees of degradation occurred. This indicated that actin was the substrate of cathepsin D, and the degree of degradation of cathepsin D was different with the degree of oxidation. Oxidation changed the conformation of cathepsin D and thus altered the degree of degradation of actin. In the non-oxidized cathepsin D and oxidized myofibrillar protein (D + OMP) treatment groups, the degree of troponin degradation decreased with increasing oxidative concentration, indicating that the concentration of H_2_O_2_ affected actin degradation and the degree of troponin degradation was inhibited at high concentrations of H_2_O_2_ (concentration greater than 0.5 mM). The high concentration of oxidation may cause actin aggregation and lead to reduced degradation. In the incubation group of oxidized cathepsin D and oxidized myofibrillar protein, that is, the OD + OMP group, actin was degraded compared with the control group. When the concentration of H_2_O_2_ was 5 mM, the degradation degree of actin was the lowest, which was reduced to 81.56% of the control group. This indicated that actin degradation was greatest when both cathepsin D and MP were oxidized by high concentrations of H_2_O_2_.

#### 2.5.4. Desmin

**Figure 9:**
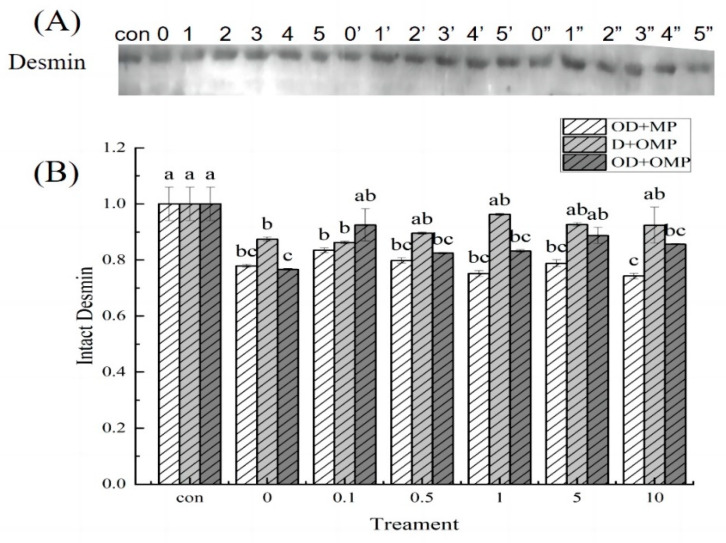
Western-blotting analysis of desmin degradation by cathepsin D at different H_2_O_2_ oxidation concentration. (**A**) Western blotting analysis of the degradation of myactin by cathepsin D in different treatment groups; (**B**) degradation of myactin in different treatment groups; 0, 1, 2, 3, 4 and 5 representing treatment with 0, 0.1, 0.5, 1, 5, 10 mM H_2_O_2_ oxidized cathepsin D and unoxidized myofibrillar protein incubation groups(OD + MP); 0′, 1′, 2′, 3′, 4′ and 5′ was the group incubated with cathepsin D and 0, 0.1, 0.5, 1, 5, 10 mM H_2_O_2_ oxidized myofibrillar protein (D + OMP); 0″, 1″, 2″, 3″, 4″ and 5” is the grouping incubated with 0, 0.1, 0.5, 1, 5, and 10 mM H_2_O_2_ oxidized cathepsin D and 0, 0.1, 0.5, 1, 5, and 10 mM H_2_O_2_ oxidized myofibrillar protein (OD + OMP). a–c: different lowercase letters denote significant differences (*p* < 0.05).

Desmin is an important cytoskeletal protein in cells, which can connect single myofibrils around the Z disc or other organelles [33]. The hydrolysis of desmin is usually considered as an indicator of skeletal muscle degradation, and the degradation of desmin has a great impact on meat quality. As shown in Figure 9A, it could be found that different incubation groups had different degrees of degradation. Cathepsin D can degrade the desmin of *Coregonus peled*, indicating that the skeletal muscle of *Coregonus peled* is degraded under the action of cathepsin D. As shown in Figure 9B, in the incubation group of oxidized cathepsin D and unoxidized protein (OD + MP), actin showed different degrees of degradation compared with the control group (freshly extracted MP). Under high concentration of oxidation, the degradation of actin was slightly higher than that under low concentration of oxidation. These results indicated that H_2_O_2_ oxidation could accelerate the degradation of myofibrillar proteins by cathepsin D. In the treatment group incubated with unoxidized cathepsin D and oxidized myofibrillar protein, that is, D + OMP, the degradation degree of desmin was first decreased and then increased, indicating that desmin itself was sensitive to hydroxyl radicals, and the change of H_2_O_2_ concentration would affect the degradation of desmin. Thereinto, the degradation degree was greater at a high concentration. In the OD + OMP group, with the different oxidation concentrations, the degree of degradation of desmin was also different, indicating that when hydroxyl radicals oxidized cathepsin D and myofibrillar protein at the same time, they altered the action site, resulting in different degrees of degradation of desmin.

#### 2.5.5. Troponin-T

**Figure 10:**
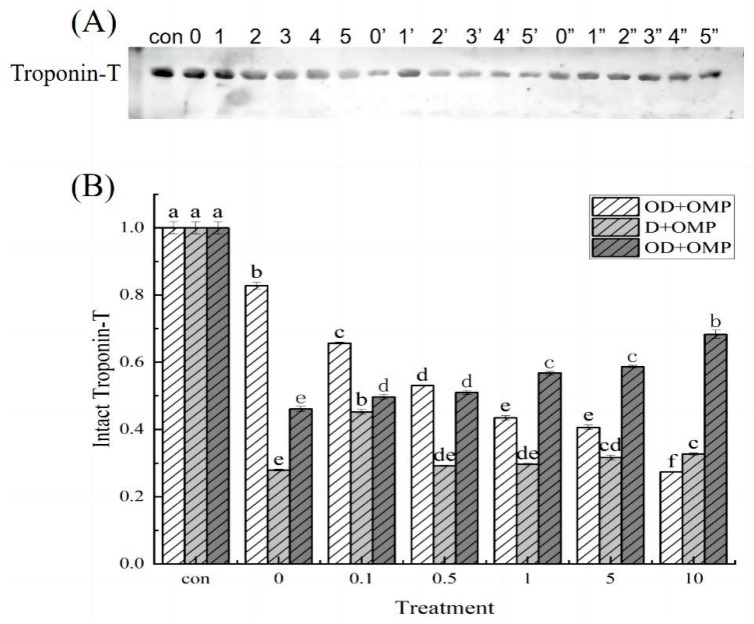
Western-blotting analysis of troponin-T degradation by cathepsin D at different H_2_O_2_ oxidation concentration. (**A**) WB analysis of degradation of troponin-T by cathepsin D in different treatment groups. (**B**) degradation of troponin-T in different treatment groups; 0, 1, 2, 3, 4 and 5 representing treatment with 0, 0.1, 0.5, 1, 5, 10 mM H_2_O_2_ oxidized cathepsin D and unoxidized myofibrillar protein incubation groups (OD + MP); 0′, 1′, 2′, 3′, 4′ and 5′ was the group incubated with cathepsin D and 0, 0.1, 0.5, 1, 5, 10 mM H_2_O_2_ oxidized myofibrillar protein (D + OMP); 0″, 1″, 2″, 3″, 4″ and 5″ is the grouping incubated with 0, 0.1, 0.5, 1, 5, and 10 mM H_2_O_2_ oxidized cathepsin D and 0, 0.1, 0.5, 1, 5, and 10 mM H_2_O_2_ oxidized myofibrillar protein (OD + OMP). a–f: different lowercase letters denote significant differences (*p* < 0.05).

Troponin is a trimeric complex composed of troponin-T, troponin -I and troponin -C. In proteomics, the degradation of troponin-T is commonly used as a marker of protein degradation [34]. As shown in Figure 10A, it could be clearly observed that the degradation of troponin-T in each incubation group was compared with the control group, and the gray scale of the degradation band changed significantly compared with the control group. As shown in Figure 10B, in the OD + MP group, the oxidized cathepsin D and unoxidized myofibrillar protein were incubated. The degradation of troponin-T showed different degrees of degradation, indicating that troponin could be degraded by cathepsin D, and the degradation degree also increased with the increase in oxidative intensity, indicating that the degradation of troponin-T by oxidized cathepsin D was affected by H_2_O_2_ oxidation. In the grouping of unoxidized cathepsin D and oxidized myofibrillar protein (D + OMP group), cathepsin D could degrade different concentrations of oxidized myofibrillar protein. The difference in oxidation concentration led to the difference in the degree of degradation of troponin-T by cathepsin D, and the degradation degree of this treatment group was higher than that of the OD + MP group. These results indicated that oxidized myofibrillar protein could be better degraded by cathepsin D. In the incubation group of oxidized cathepsin D and oxidized myofibrillar protein, that is, the OD + OMP group, there were different degrees of degradation of troponin-T. With the increase in oxidation degree, the degradation degree of troponin-T gradually decreased, and the structure of cathepsin D and myofibrillar protein was changed by oxidation. The degree of degradation of the treated group showed different results with different oxidation concentrations, indicating that H_2_O_2_ could change the activity of protease and the structure of protein, so that the degree of proteolytic degradation of myofibrillar protein also changed.

## 3. Materials and Methods

### 3.1. Sample Descriptions

All *Coregonus peled* used in this study were raised under the same feeding and management conditions (weight: 1000 ± 50 g; length: 30 ± 2.5 cm) at a commercial fishery processing company (SaiHu Fishery Technology Development Co., Ltd., Bole, China). Freshly caught *Coregonus peled* were transported to the laboratory within 5 h by cold-chain transportation. The dorsal muscles were cut into blocks by removing the hemoglobin, frozen immediately in liquid nitrogen, and rapidly stored at −80 °C.

### 3.2. Preparation of Myofibrillar Protein (MP)

Myofibrillar protein (MP) was extracted following the method described previously [35] with slight modifications. Five grams of fish dorsal muscle was added to pre-cooled deionized water (1:10, *w*/*w*) and homogenized by a homogenizer (Scientz Co., Ningbo, China) at 10,000 rpm for 1 min until the muscle was broken (to avoid air bubbles). The homogenate was then centrifuged at 8000× *g* for 10 min (Thermo Fisher Technologies, Waltham, MA, USA), and the supernatant was discarded to collect the precipitate. The precipitate was washed by adding 10 times the volume of the precipitate in 0.3% NaCl solution and centrifuged again at 8000× *g* for 10 min, then the supernatant was discarded. Next, the precipitate was dissolved in a 10-fold volume of pre-cooled 0.6 M NaCl-Tris-HCl buffer (pH 7.0). After the supernatant was completely dissolved, it was homogenized with pre-cooled deionized water (1:4, *v*/*v*) and centrifuged at 8000× *g* for 10 min at 4 °C to obtain the precipitate. The final precipitate was resuspended in 0.6 M phosphate buffer (pH 7.0), the insoluble material was removed by filtration through three layers of gauze, and the filtrate was used as MP. The concentration of MP was measured using the biuret method [36] with bovine serum albumin as standard.

### 3.3. Preparation of the Hydroxyl Radical Oxidation System

The active and pure cathepsin D (C8696, Sigma, St. Louis, MO, USA, from the human liver) was subjected to a hydroxyl radical oxidation according to the method of Park et al. [37]. This system is mainly produced by hydrogen peroxide, ascorbic acid, and ferric chloride through the redox reaction of iron, which can also be called the hydroxyl radical oxidation system. The reaction process is Vitamin C (Vc) + Fe^3+^→Fe^2+^, Fe^2+^ + H_2_O_2_→OH. This oxidation system contained 0.1 mM Vc, 0.01 mM FeCl_3_ and different concentrations of H_2_O_2_ (0, 0.1, 0.5, 1, 5 and 10 mM) in 50 mM phosphate buffer (pH 6.0). The samples were then incubated for 1 h at 4 °C. After incubation, oxidation was terminated by the addition of Ethylene diamine tetraacetic acid (EDTA)/Ethylene diamine tetraacetic acid disodium salt (EDTA-2Na) at a final concentration of 1 mM. The oxidation system of myofibrillar protein was the same as that of cathepsin D, and the final concentration of myofibrillar protein was 10 mg per milliliter.

### 3.4. Measurement of the Activity of Cathepsin D

Cathepsin D activity was measured using acidified bovine serum protein as a substrate according to the method of Minarowska et al. [38]. First, 100 μL of bovine hemoglobin containing 2.5% (*w*/*v*) was mixed with 50 μL citrate buffer (contains citric acid and sodium citrate, pH 2.8) and incubated at 37 °C for 30 min for denaturation, and 25 μL of the enzyme system was added and incubated at 37 °C for 60 min. The reaction was finished by adding 50 μL of 15% (*w*/*v*) trichloroacetic acid. The TCA soluble polypeptide in the supernatant was released by the enzymatic reaction and centrifuged at 2000× *g* for 5 min. The content of TCA soluble polypeptide was determined by the method of Lowry et al. [39]. The relative activity of cathepsin D under different oxidation concentrations was determined by using the TCA soluble polypeptide at 0 mM H_2_O_2_ concentration as control.

### 3.5. Measurement of the Carbonyl Content of Cathepsin D

According to the method of Oliver et al. [40], the carbonyl content was determined by the reaction of UV-vis spectrophotometer (Cary 50, Shanghai Spectrum Instrument Co., Ltd., Shanghai, China) with 2,4-dinitrophenylhy-drazine (DNPH): 70 µL of the sample (difference method) was incubated with 1 mL 10 mM DNPH in the dark at 25℃ for 1 h, and then myofibrillar protein was precipitated with 1 mL of 20% trichloroacetic acid. The mixture was then centrifuged at 8000× *g* for 5 min at 4 °C, the supernatant was discarded, and the precipitate was dissolved in 1 mL absolute ethanol-ethyl acetate (1:1, *v*/*v*) and washed 3 times to remove free DNPH. The obtained precipitate was finally dissolved in 3 mL of a solution containing 6 M guanidine hydrochloride and kept in a 37 °C water bath for 10 min to dissolve the precipitate. The solution was then centrifuged under the conditions described above and the supernatant was retained. The carbonyl content was determined by the molar extinction coefficient of 22,000 M^−1^ CM^−1^ at 370 nm, and the carbonyl content of the 0 mM H_2_O_2_ treatment group was set to 1, and then the relative content was calculated for the remaining groups.

### 3.6. Determination of the Sulfhydryl Content of Cathepsin D

Following the method of Qin et al. [41] with slight modifications, 4 mL of 0.1 M phosphate buffer (containing 8 M urea, 3% SDS, pH 6.0) was added to 1 mL of the sample mixture and then incubated with 0.1 mL of 10 mM 5,5′-Dithiobis(2-nitrobenzoic acid) (DTNB) at 25 °C for 15 min. Absorbance was measured at 412 nm and calculated using a molar extinction coefficient of 13,600 M^−1^ CM^−1^. Moreover, the carbonyl content of the 0 mM H_2_O_2_ treatment group was set to 1, and the relative content was calculated for the remaining treatment groups.

### 3.7. Measurement of the Intrinsic Fluorescence of Cathepsin D

The intrinsic fluorescence spectroscopy of oxidized cathepsin D was performed by the method described previously [1] with minor modifications. The emission spectra between 300 and 400 nm was obtained using a fluorescence spectrophotometer at an excitation wavelength of 283 nm.

### 3.8. Circular Dichroism (CD) Analysis of Cathepsin D

The secondary structure of oxidized cathepsin D was studied using a MOS-450 circular dichroism spectrometer (Biologic, Rennes, France) and a quartz absorption cell with an optical path length of 1 mm [42]. The scanning wavelength was 190–240 nm.

### 3.9. Ultraviolet (UV) Absorption of Cathepsin D

The UV spectroscopy were performed according to the previously reported methods [43]. The UV absorption spectra of all the samples were scanned at 230–500 nm range and then analyzed using a UV-visible spectrophotometer at room temperature.

### 3.10. Incubation of the Cathepsin D with the MP

To determine whether the degradation of MP by oxidized cathepsin D is different, we incubated both oxidized and unoxidized MP samples with known quantities of oxidized and unoxidized cathepsin D [44]. In brief, 1 mL (2 mg/mL) of MP was added into a reaction medium containing 65 µL of oxidized or unoxidized cathepsin D. The reaction system was incubated at 25 °C for 2 h, and then 0.5 mL of 100 mmol/L EDTA was added to stop the reaction. Next, an equal volume of loading buffer was added and mixed well. After blending, the samples were heated in water at 100 °C for 5 min for subsequent sodium dodecyl sulfate-polyacrylamide gel electrophoresis and western blotting analysis.

### 3.11. SDS-PAGE and Western Blotting Analyses

The degradation of MP by oxidized cathepsin D was evaluated by SDS-PAGE and WB according to the method of Laemmli [45] with slight modifications. The degradation level of troponin-T, desmin, and actin was detected through SDS-PAGE with 15%, 15% and 12% polyacrylamide separating gels, respectively 5% polyacrylamide stacking gels were used, 10 µg samples were loaded per well for desmin and troponin-T, and 7 µg of samples were loaded per well for actin. The sample was loaded into the lane on the gel using the Bio-Rad Mini-Protean II system (Bio-Rad Laboratories, Hercules, CA, USA); the samples were run at 80 V of 30 min for the 5% stacking gel and 120 V of 1 h for separating gel until the leading edge of the front was at the bottom of the gels. The gel was then stained using coomassie brilliant blue R-250 for 30 min and then decolorized overnight until the solution became clear. The target proteins were simultaneously transferred onto polyvinylidene difluoride (PVDF) membranes through a semi-dry transfer device (Bio-Rad Laboratories, Hercules, CA, USA) at 200 mA for 30 min. Afterwards, the membrane was blocked with 5% skimmed milk powder in Tris-buffered saline containing 0.05% Tween 20 (TBST) at room temperature for 1 h with gentle shaking. Then, the membranes were incubated with primary antibodies against anti-actin mouse monoclonal antibody (3E9, abbkine, CA, USA), anti-desmin mouse monoclonal antibody (ab8470, abcam, Cambridge, UK), and anti-troponin-T monoclonal antibody (ab10214, abcam, Cambridge, UK) diluted in TBST at 4 °C overnight. After incubation, the membrane was washed with TBST three times for 15 min each and rinsed with tris-buffered saline (TBS) for 15 min, then incubated with the goat anti-mouse IgG H&L (HRP) conjugated secondary antibody (ab6789, abcam, Cambridge, UK) at indoor temperature for 1 h. Thereinto, the membrane was washed four times in TBST for 15 min each time. The target proteins were illuminated by using the Clarity Western ECL detection kit (Cambridge, UK), and the images were imaged and analyzed by Quantity One software (Bio-Rad Laboratories, Hercules, CA, USA).

### 3.12. Statistical Analysis

All experiments were completed in triplicate, and statistical analysis was performed by SPSS 25.0 (SPSS Inc., Chicago, IL, USA). Analysis of variance (ANOVA) was used to measure the significance of the major influences, and significant differences between the means were determined by Duncan’s multiple range test. The significance level was set as *p* < 0.05.

## 4. Conclusions

As the oxidative concentration increased, the enzyme structure of the tryptophan residue gene of cathepsin D changed, resulting in fluorescence quenching and a decrease in the fluorescence absorption intensity of cathepsin D. The UV absorption intensity of cathepsin D also decreased with increasing oxidation intensity, indicating that cathepsin D was sensitive to hydroxyl radicals and that the protein’s primary structure had changed. Hydroxyl radical oxidation altered the tertiary structure of cathepsin D, as measured by CD.

Furthermore, when oxidized/unoxidized cathepsin D was incubated with oxidized/unoxidized myofibrillar protein, it was found that oxidized cathepsin D was more effective in degrading the MHC of oxidized myofibrillar protein than unoxidized myofibrillar protein. However, the degradation of actin, myactin, and troponin varied with different treatments. In practice, protease, oxidation, and protein degradation are highly complex processes that involve many intricate physiological changes and require further exploration and study.

## Data Availability

The data presented in this study are available on request from the corresponding author.
